# Glandular Odontogenic Cyst in Dentigerous Relationship: An Uncommon Case Report

**DOI:** 10.1155/2019/8647158

**Published:** 2019-07-04

**Authors:** Jean Carlos Barbosa Ferreira, Eneida Franco Vêncio, Rodrigo Tavares de Sá, Giovanni Gasperini

**Affiliations:** ^1^Department of Oral and Maxillofacial Surgery, Clinical Hospital, Federal University of Goiás, Goiânia, Brazil; ^2^Department of Oral Medicine (Oral Pathology), Dental School, Federal University of Goiás, Goiânia, Brazil

## Abstract

Glandular odontogenic cyst (GOC) is an uncommon cyst of the jaw. Less than 200 cases are reported in the literature, and only 22 cases are associated with an unerupted tooth (dentigerous relationship). Although it is an asymptomatic lesion, it can be destructive and has high recurrence rates. The diagnosis can be especially challenging due to the lack of distinct diagnostic clinic-radiological criteria and nonspecific microscopic features, mimicking benign and malignant lesions. Conservative surgical treatment has been the choice for most surgeons, but marginal or partial jaw resection has been reported. This report describes a rare case of GOC in a dentigerous relationship, which was treated with enucleation and peripheral osteotomy.

## 1. Introduction

Glandular odontogenic cysts (GOCs) are uncommon jawbone cysts of odontogenic origin which were firstly described in 1987 by Padayachee and Van Wyk [1] as a “botryoid” odontogenic cyst with glandular component and denominated “sialo-odontogenic cyst.” Gardner et al. [2] in 1988 established this cyst as a distinct entity-denominated glandular odontogenic cyst, which was classified as an odontogenic cyst by the WHO in 1992 [3].

To the best of our knowledge, there are 196 GOCs in the English literature [4–14]. Clinically, GOCs are small and usually appear as an asymptomatic swelling, though a few cases have presented with pain and paresthesia. The most common site is the mandible, particularly the anterior region. The cyst shows no sex predilection and mostly affects middle-aged individuals, between 45 and 50 years old; however, there are also reports in pediatric patients [4, 15]. According to Kaplan et al. [16], its recurrence rate is around 35.9%, particularly when conservative surgical treatment is chosen.

Radiographically, it presents as a uni- or multilocular cystic lesion, with well-defined margins, though some lesions exhibit scalloped borders. Other findings include loss of cortical integrity, root resorption, and association with unerupted teeth [15]. Some cases show a dentigerous, lateral periodontal, and “globulomaxillary” relationship [17].

Microscopically, the diagnosis of GOC can be challenging, given the rarity of the lesion and the fact that the differential diagnosis includes benign and malignant lesions, such as botryoid cysts, surgical ciliated cysts, radicular or dentigerous cysts with metaplastic changes, and low-grade mucoepidermoid carcinoma (MEC) [14, 16, 17]. Histopathological features for the GOC have been described, but the exact microscopic criteria necessary for diagnosis have not been universally accepted. These features include a nonkeratinized stratified squamous lining epithelium with focal thickening (plaques) in the cystic lining, eosinophilic cuboidal or ciliated columnar cells, mucous cells, and interepithelial gland-like structures [1–3, 16].

Several treatment modalities have been indicated for the GOCs, including conservative approaches, such as enucleation with or without curettage, marsupialization, peripheral ostectomy and chemical cauterization with Carnoy's solution, and marginal resection/partial jaw resection [4]. This report documents an uncommon case of GOC in a dentigerous relationship (GOC-DR), which was treated with enucleation and peripheral osteotomy.

## 2. Case Report

**Figure 1:**
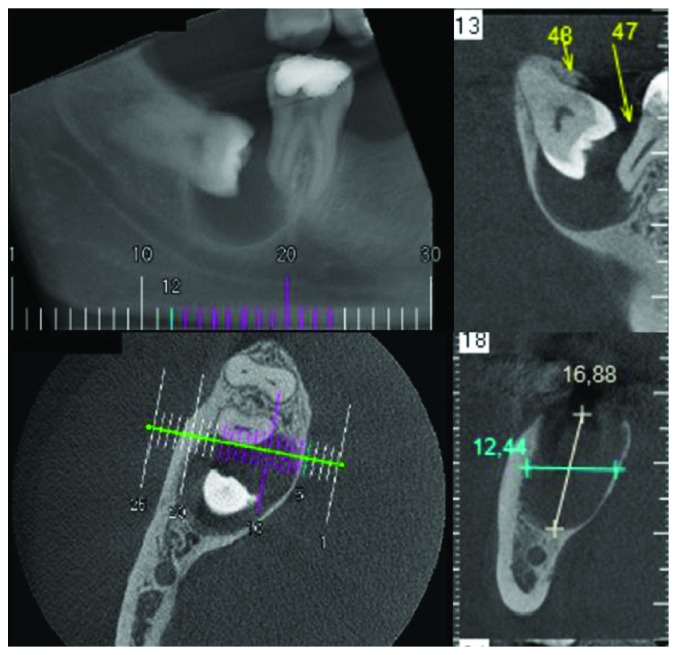
Preoperative CBCT showing well-defined unilocular radiolucency associated with an impacted right third molar extending to the distal root of the second molar.

A 36-year-old male, with no medical history, was referred to the Clinical Hospital of the Federal University of Goiás, Goiânia, Goiás, Brazil, for evaluation of an asymptomatic radiolucent lesion in the posterior mandible region. Cone-beam computed tomography (CBCT) scan showed a well-defined unilocular radiolucency associated with an impacted right third molar, extending to the distal root of the second molar, measuring 17 × 12.5 mm (Figure 1). Intraoral examination revealed signs of healthy gingiva; absence of teeth 16, 36, 37, and 46; and absence of bone expansion. However, clinical attachment loss in the distal root of tooth 47 with pulp vitality was verified. Previous aspiration was negative and previous diagnosis of dentigerous cyst was made. Due to the small size of the lesion, the treatment choice included tooth removal, enucleation, and peripheral osteotomy. A thick cystic wall was evident during the surgical procedures.

**Figure 2:**
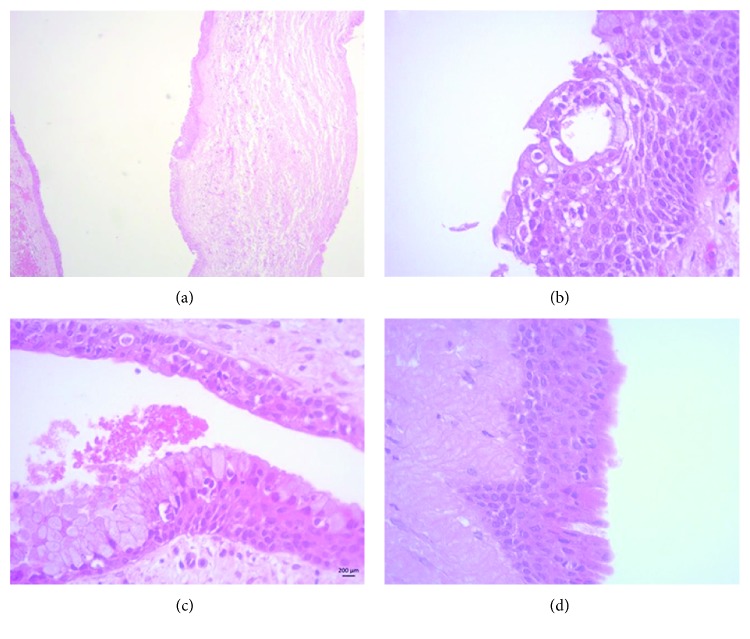
Microscopic features of the GOC. (a) Cystic cavity lined by nonkeratinized stratified squamous epithelium of varying thickness. (b) Duct-like structures observed in the cystic lining. (c) Presence of numerous goblet cells. (d) Note the presence of eosinophilic cuboidal cells. Slides stained with haematoxylin and eosin. Original magnifications: 50x and 400x.

The histopathological examination revealed cyst wall lining by nonkeratinized stratified squamous epithelium with varied thickness (Figure 2(a)). Duct-like structures surrounded by cuboidal cells and numerous mucous cells were also identified (Figures 2(b) and 2(c)). The superficial layer of the epithelium showed columnar ciliated and eosinophilic cuboidal cells, also called “hobnail cells” (Figure 2(d)).

**Figure 3:**
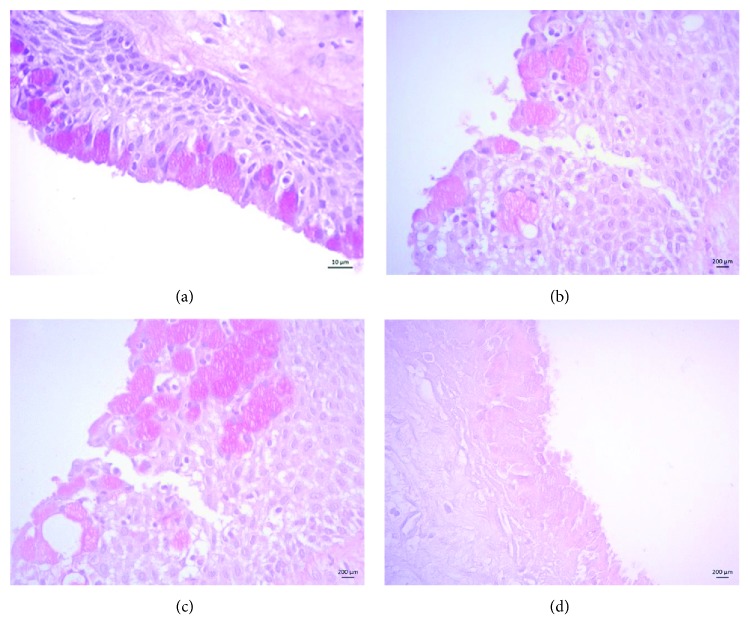
Special stains for GOC. (a, b) Periodic acid-Schiff- (PAS-) and periodic acid-Schiff diastase- (PAS-D-) positive goblet cells showing glycogen-rich cells. (c) Note the glandular-like structure stained by PAS-D. Mucin-secreting cells were also identified.

**Figure 4:**
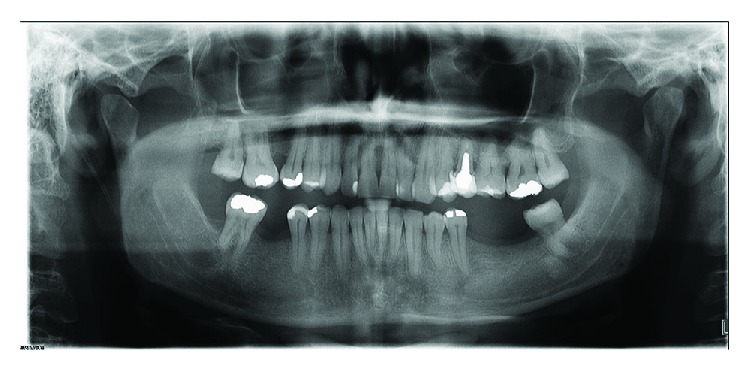
Patient's orthopantomogram (OPG) showing bone healing after a 2-year follow-up.

Glycogen-rich and mucin-secreting cells were highlighted by periodic acid-Schiff (PAS), periodic acid-Schiff diastase (PAS-D) (Figures 3(a)–3(c)), and mucicarmine staining (Figure 3(d)). A final diagnosis of GOC was made following the criteria established by Fowler et al. [17]. The postoperative orthopantomogram (OPG) revealed no recurrence one year postsurgery (Figure 4).

## 3. Discussion

Our study reports an uncommon case of GOC associated with an unerupted third molar mimicking a dentigerous cyst. This characteristic was defined by Fowler et al. [17] as a “dentigerous relationship.” In the English literature, only 22 similar cases have been documented [18–22].

Table 1 [17–27] summarizes previous published cases of GOC-DR. Complete clinical data was not be available in all cases. Males were more often affected (male : female ratio, 3 : 1) and age ranged from 21 to 62 years old (mean 38 years old). The mandible was affected in 53.8% of cases, in which 57.1% involved the unerupted third molar and 42.8% the canine. Swelling was the most common clinical presentation with 85.7%, followed by pain (28.5%), and numbness (14.2%). Unilocular radiolucency was described in 10 cases (76.9%). Half of the cases were treated with enucleation, followed by curettage (41.6%) and block resection (8.3%). In the present case, a 36-year-old male presented an asymptomatic mandibular lesion detected incidentally by routine radiological examination treated with enucleation and peripheral osteotomy [4, 16, 18, 21, 22]. It should be noted that unlike classic GOC, GOC-DR has a strong predilection for the male sex and posterior mandible.

Clinical diagnosis of GOC is challenging. The differential diagnosis includes radicular and dentigerous cysts, odontogenic keratocysts, and ameloblastoma. Although Krishnamurthy et al. [19] suggest that a preoperative aspiration biopsy may be helpful in diagnosing GOC, in our case, it was negative, as reported by Momeni Roochi et al. [23]. Distinct fluids have been reported in the literature, including clear with low viscosity, creamy high-viscosity, and brownish-red liquids [19, 23, 28, 29]. Another interesting clinical finding in our case was the presence of a thick cystic wall, contrary to findings shown by Thor et al. [30].

The histopathological diagnosis of GOC also remains a challenge. Microscopic features include focal epithelial thickening, epithelial plaques, and glycogen-rich epithelial cells, which are also observed in botryoid and lateral periodontal cysts. The presence of ciliated epithelium and duct-like spaces with mucous cells and eosinophilic cuboidal cells located in the epithelial surface support the diagnosis of GOC [17, 31]. According to Fowler et al. [17], the presence of microcysts, clear cells, and epithelial spheres may be helpful in distinguishing GOC-DR from dentigerous cyst with metaplastic changes. The most important and difficult distinction according to Kaplan et al. [16] is the differentiation of low-grade MEC from GOC, especially its multicystic variant. Ciliated cells, superficial cuboidal cells, epithelial whorls, and intraepithelial microcyst or duct-like structures are not typical for low-grade MEC, which can help in the differentiation. Immunostain for MASPIN, Ki-67, and CKs 18 and 19 may be helpful to distinguish GOC from low-grade MEC [14].

Due to the overlapping of histological features with others lesions, Fowler et al. [17] suggested 10 microscopic parameters for diagnosing GOC: surface eosinophilic cuboidal cells or “hobnail cells”, intraepithelial microcysts or duct-like spaces lined by a single layer of cuboidal to columnar cells, apocrine snouting of hobnail cells, clear or vacuolated cells, variable thickness in the cyst lining, papillary projections or “tufting” into the cyst lumen, mucous goblet cells, epithelial spheres, or plaque-like thickening cilia, and multiple compartments. According to the authors, the presence of seven or more microscopic parameters is highly predictive of a diagnosis of GOC. In our case, only multiple compartments and papillary projections were not evidenced.

Minor surgical procedures, such as enucleation with or without curettage and peripheral ostectomy, are the most common treatment modalities reported in the literature [4, 32]. In this study, enucleation associated with peripheral osteotomy was performed due to three factors: patient choice, clinical and radiological diagnosis of a dentigerous cyst, and lesion size (17 × 12.5 mm). On the other hand, radical treatments, such as marginal resection, can sometimes be considered due to the biological behavior of GCO, particularly due to local aggressiveness and recurrence rates around 21-55% [15, 19, 33]. Some reports suggest that recurrence is more common in larger lesions, with cortical bone perforation and multilocular radiographic appearance [30, 32]. In the present case, neither of these characteristics was evident and no recurrence was detected after two years follow-up.

## 4. Conclusion

This report describes an uncommon case of GOC-DR mimicking other lesions in the oral cavity. These lesions tend to most commonly affect the posterior mandible and younger male patients.

## Figures and Tables

**Table 1:** Clinical and radiological data of GOC in dentigerous relationship.

Year	Author	Number of cases	Age/gender	Site	Clinical presentation	Radiologic features	Treatment	Follow-up (year)
2019	Ferreira et al. (our case)	1	36/M	Mandibular right third molar	Asymptomatic	Unilocular radiolucency	Enucleation/peripheral osteotomy	1
2015	Momeni Roochi et al. [23]	1	62/M	Mandibular right canine impacted	Swelling	Unilocular radiolucency	Enucleation	3
2012	Cano et al. [18]	1	54/M	Mandibular right third molar (ramus/body)	Swelling	Multilocular radiolucent, large and well-defined	Enucleation and curettage, reconstruction	3
2011	Fowler et al. [17]	8	NS	NS	NS	NS	NS	NS
2009	Krishnamurthy et al. [19]	1	21/M	Mandibular left third molar	Swelling	Multilocular radiolucency	En bloc resection	2
2006	Kasaboglu et al. [24]	1	45/M	Mandibular left canine	Swelling and numbness	Unilocular radiolucency with a well-defined border	Enucleation	0.5
2006	Shen et al. [25]	2	Case 1: 40/M; case 2: NS	Case 1: maxillary tooth-like structures; case 2: NS	Case 1: NS; case 2: NS	Case 1: unilocular radiolucency; case 2: NS	Case 1: NS; case 2: NS	NS
2006	Yoon et al. [20]	1	66/F	Mandibular right third molar	Swelling and painful	Unilocular radiolucency, thin sclerotic margin, root resorption	Enucleation	1
2005	Qin et al. [26]	5^∗^	Case 1: 28/M; case 2: 40/M; case 3: 25/M; case 4: 22/M; case 5: 52/F	Case 1: maxilla L (21-27); case 2: maxilla R (11-16); case 3: maxilla R (13-16); case 4: maxilla L (21-23); case 5: maxilla (16-25)	Case 1: NS; case 2: NS; case 3: NS; case 4: NS; case 5: NS	Case 1: Unilocular radiolucency; case 2: unilocular radiolucency, irregular borders; case 3: unilocular radiolucency, irregular borders; case 4: unilocular radiolucency; case 5: multilocular radiolucency	Case 1: curettage; case 2: curettage; case 3: curettage; case 4: curettage; case 5: curettage	NS
2005	Kaplan et al. [21, 22]	1	49/M	Mandibular left third molar	Swelling and painless	Unilocular radiolucency	Enucleation, peripheral ostectomy, reconstruction with iliac crest & alloplast bone graft	4
1996	Ide et al. [27]	1	54/F	Mandibular right canine	Asymptomatic	Unilocular radiolucent definite contained the crown of the horizontally impacted right canine	Enucleation	1

Legend: NS: not specified; M: male; F: female; L: left; R: right. ^∗^Case 2 shown in the work of Shen et al. [25].
